# Collimated Microbeam Reveals that the Proportion of Non-Damaged Cells in Irradiated Blastoderm Determines the Success of Development in Medaka (*Oryzias latipes*) Embryos

**DOI:** 10.3390/biology9120447

**Published:** 2020-12-05

**Authors:** Takako Yasuda, Tomoo Funayama, Kento Nagata, Duolin Li, Takuya Endo, Qihui Jia, Michiyo Suzuki, Yuji Ishikawa, Hiroshi Mitani, Shoji Oda

**Affiliations:** 1 Department of Integrated Biosciences, Graduate School of Frontier Sciences, The University of Tokyo, Chiba 277-8562, Japan; nagata.kento@qst.go.jp (K.N.); 3609676058@edu.k.u-tokyo.ac.jp (D.L.); 7000984892@edu.k.u-tokyo.ac.jp (T.E.); 9541072172@edu.k.u-tokyo.ac.jp (Q.J.); mitani@edu.k.u-tokyo.ac.jp (H.M.); odasho@edu.k.u-tokyo.ac.jp (S.O.); 2 Takasaki Advanced Radiation Research Institute, Quantum Beam Science Research Directorate, National Institutes for Quantum and Radiological Science and Technology (QST), Gunma 370-1292, Japan; funayama.tomo@qst.go.jp (T.F.); suzuki.michiyo@qst.go.jp (M.S.); 3 National Institute of Radiological Sciences, Quantum Medical Science Directorate, National Institutes for Quantum and Radiological Science and Technology (QST), Chiba 263-8555, Japan; y.ishikawa@sea.plala.or.jp

**Keywords:** blastoderm, medaka, microbeam irradiation, brain damage, teratogenesis, embryogenesis, pre-implantation period

## Abstract

**Simple Summary:**

Studies on teratogenesis in mammals have revealed that exposure to ionizing radiation (IR) during the pre-implantation period induces a high frequency of lethality instead of teratogenesis. Here, to elucidate the IR-induced disturbance of embryonic development when IR exposure occurs during the pre-implantation period, we utilized medaka as a vertebrate model for clear observation of developmental process for its transparency. Blastula embryos exposed to IR with a lower lethal dose (gamma-rays) transiently exhibited smaller brains than those of sham-controls, however, their brain size restored equally to those of controls until hatching. We then conducted targeting irradiation, which allowed various proportions of blastoderm cells to be exposed to IR (carbon-ions), and identified that the loss of approximately 10% or less of blastoderm was compensated by the remaining non-damaged blastoderm cells even though they transiently exhibited smaller brains. In contrast, when IR exposure occurred during the late embryogenesis period, 3 days post fertilization, the brain size was not completely restored until hatching even with a lower lethal dose. Collectively, blastoderm cells with IR-induced injury undergo transient delays in brain development, however, can avoid teratogenesis at hatching presumably for their pluripotency whereas embryos during the late embryogenesis period lack the ability to do so.

**Abstract:**

It has been widely accepted that prenatal exposure to ionizing radiation (IR) can affect embryonic and fetal development in mammals, depending on dose and gestational age of the exposure, however, the precise machinery underlying the IR-induced disturbance of embryonic development is still remained elusive. In this study, we examined the effects of gamma-ray irradiation on blastula embryos of medaka and found transient delay of brain development even when they hatched normally with low dose irradiation (2 and 5 Gy). In contrast, irradiation of higher dose of gamma-rays (10 Gy) killed the embryos with malformations before hatching. We then conducted targeted irradiation of blastoderm with a collimated carbon-ion microbeam. When a part (about 4, 10 and 25%) of blastoderm cells were injured by lethal dose (50 Gy) of carbon-ion microbeam irradiation, loss of about 10% or less of blastoderm cells induced only the transient delay of brain development and the embryos hatched normally, whereas embryos with about 25% of their blastoderm cells were irradiated stopped development at neurula stage and died. These findings strongly suggest that the developmental disturbance in the IR irradiated embryos is determined by the proportion of severely injured cells in the blastoderm.

## 1. Introduction

It has been widely reported in mammal models that prenatal exposure to ionizing radiation (IR) can interfere with embryonic and fetal development, depending on the dose and gestational age at which the IR exposure occurs [1,2,3]. Since mouse fetuses have to be sacrificed to identify the external malformations, it was hard to detect them by sequential observations during embryogenesis. Like zebrafish medaka is a model organism extensively used in many fields of life science research. Medaka is an oviparous species and the embryos are highly transparent so that the process of embryonic development can be observed clearly under a light microscope, which enables us to recognize abnormalities clearly during their embryogenesis [4,5].

Medaka embryonic stem (ES) cell lines have been established from blastoderm cells and it has been shown that the medaka ES cells are capable of giving rise to various differentiated cell types in vitro [6]. ES cells transplanted into blastula embryo contributed to develop various tissues and organs in vivo to indicate the pluripotency of the blastoderm cells in medaka [7,8]. However, the precise machinery for restoration in blastoderm cells after exposure to IR are remained elusive. In this study, we examined the effects on embryonic development of gamma-ray irradiation on blastula embryos of medaka. In addition, we conducted targeted irradiation at the center part of blastoderm in medaka embryos with a collimated carbon-ion beams with a diameter of 70, 120, and 180 μm to irradiation of about 4, 10 and 25% of the blastoderm cells at the facility of Takasaki Ion Accelerators for Advanced Radiation Application (TIARA) in National Institutes of Quantum Beam Science and Technology (QST), and observed embryonic development until hatching (7 days post-fertilization). The collimated carbon-ion microbeam irradiation facility of TIARA enables us to locally irradiate a very small region of tissue or even a single cell [9], which is useful for research on radiation biology [9,10,11]. In our previous study, we have already established the procedures of targeted irradiation to the right half-hemisphere of the optic tectum (OT) in the medaka embryonic brain at stage 28 (3 days post fertilization) with collimated carbon-ion beam (250 μm diameter), which induced apoptotic cells reliably and reproducibly in the locally irradiated region [12].

In this study, to reveal the disturbance of embryonic development after exposure to IR in extremely early embryogenesis period, blastula embryos in medaka, we analyzed the effects on embryonic development of gamma-ray irradiation and targeted irradiation of carbon-ions at the center part of blastoderm with different diameters of microbeam which allowed various proportions of blastoderm cells to be exposed to IR (carbon-ions).

## 2. Materials and Methods

### 2.1. Ethics

This research was conducted using protocols approved by the Animal Care and Use Committee of the University of Tokyo (permit number: C-09-01, on 19 May 2009; C-14-02, on 25 June 2015) and the Animal Care and Use Committee of the Takasaki Advanced Radiation Research Institute, QST (QST-Takasaki) (permit number: 16-T003, on 10 June 2016). The 0.02% (w/v) tricaine methanesulfonate (MS222) (Merck KGaA, Darmstadt, Germany) was used as anaesthesia and the efforts made to minimize suffering to the animals during anaesthesia and surgery would be appropriate.

### 2.2. Fish and Embryos

Hd-rR inbred strain medaka (*Oryzias latipes*) [13] were used. The fish were kept at 26–28 °C under a 14-h light and 10-h dark cycle and were fed brine shrimp (*Artemia franciscana*) and powdered diet (TetraMin, Tetra Werke, Melle, Germany) three times per day. Egg clusters were collected and rubbed between two small pieces of paper towel to remove filaments on the chorion; the isolated eggs were then incubated in a Petri dish filled with 7 mL of tap water containing 0.00001% (w/v) methylene blue at 26–28 °C. The collected eggs at 8 h and 72 h after incubation were examined the development according to Iwamatsu [14] under stereomicroscope (Leica M125, Nussloch, Germany). We selected the blastula embryos at the stage 11 (8 h post-fertilization) and the late embryos at the stage 28 (72 h post-fertilization). In previous our studies, we confirmed the optically opaque cells were apoptotic cells by histological examinations [4,15]. For evaluation of brain development in irradiated embryos, we examined under a stereomicroscope (Leica M125) to determine whether developmental delay and optically opaque cells were induced several times a day for 7 days until hatching.

### 2.3. Irradiation

For X-ray irradiation, embryos at one cell stage were irradiated with X-ray machine in QST-NIRS (Chiba) (Shin-Ai-250-7, Shimadzu, Kyoto, Japan) at a dose rate 0.847 Gy/min with 200 kV, 20 mA and using 0.5 mm-Cu and 0.5 mm-Al as filters. For gamma-ray irradiation, embryos at blastula stage were irradiated with gamma-rays in The University of Tokyo emitted from ^137^Cs (Gammacell 3000Elan, MDS Nordion, Ottawa, ON, Canada) at a dose rate of 7.3 Gy/min at room temperature in a plastic tub with water. We conducted targeted irradiation of blastoderms by using a collimated carbon-ion microbeams with diameters of 70, 120 and 180 μm. We employed the collimating microbeam irradiation system [9,10] and used ^12^C^6+^ particles (26.7 MeV/u, LET = 78 keV/µm at surface of embryo) accelerated from the azimuthally varying field cyclotron installed at the Takasaki Ion Accelerators for Advanced Radiation Application (TIARA) facility of QST-Takasaki (Gunma). The irradiation procedure was essentially followed the method established for animals previously [12,16,17], with slight modifications. At first, an embryo placed on a Petri dish covered with an ultra-thin polyimide film (7.5 µm thick, Kapton^®^ film, Toray, Tokyo, Japan) was located on a custom-made aluminum frame for the microbeam-irradiation apparatus. The embryo on a sample flame was placed on a microscope stage of irradiation apparatus, then a single position of blastoderm center was targeted under microscopy. Subsequently, the embryo was irradiated with defined number of carbon ions one by one, calculated to deposit a desired absorbed dose (Gy) at each beam size. For example, 3000 carbon ions correspond to 10 Gy using a 70 μm-diameter micro-aperture (beam exit) and 140,000 carbon ions correspond to 50 Gy using a 180 μm-diameter beam exit. Targeted irradiation to an embryo finished within a few minutes. The carbon-ion beam track on a 100 μm thin film of the ion-track detector CR-39 (Solid State Nuclear Track Detector HARZLAS TNF-1, Fukuvi Chemical Industry, Fukui, Japan) at each beam size (diameter of beam exit) was confirmed before irradiation by etching the ion track on a CR-39 film (Figure S1).

### 2.4. Immunohistochemistry

For whole-mount immunostaining, chorions were removed by hatching enzyme for 20 min at room temperature. The samples were washed in PBS and were fixed in 4% paraformaldehyde in 0.1 M phosphate buffer for 3 h. For immunohistochemical analyses, fixed embryos were incubated with polyclonal anti-phospho-histone H3 (Ser10) (anti-P-H3) antibody (1:200, Merck KGaA, Darmstadt, Germany), for 1 day at 4 °C, thereafter the samples were washed with PBS and further incubated with secondary antibodies conjugated with Alexa-488 (Invitrogen, Carlsbad, CA, USA, 1:1000) for 3 h for fluorescent images and were counterstained with DAPI for 1 hr. Fluorescent images were obtained using a confocal microscope (FluoView FV1000; Olympus, Tokyo, Japan). Photomicrographs of the samples were taken with a digital camera (DFC7000T, Leica Microsystems, Wetzlar, Germany) equipped with a fluorescence microscope (BX50, Olympus, Tokyo, Japan) and anti-P-H3-labeled cells were counted.

### 2.5. RNA Isolation, Real-Time qPCR and RNA-Seq Analysis

Total RNA was isolated using ISOGEN (Nippon Gene Co., Toyama, Japan), cDNA synthesis, and quantitative real-time PCR (SYBR^®^ Premix Ex Taq™ in a Smart Cycler^®^II System, Takara Bio Inc., Kusatsu, Japan) were performed following the manufacturer’s instructions. The sequence of the medaka gene encoding p21 was obtained from the Ensembl Genome Browser (http://asia.ensembl.org/index.html) database. The primer pair of p21 forward, 5′–CAACGTGGAG AAAACACCAG–3′, and reverse, 5′–CCATTCGTCGTTTAGCTTGG–3′, were used for quantitative real-time PCR. The δδCt method was used to determine the relative expression of mRNAs in control and normalized to β-actin mRNA level [18]. RNA sequencing was performed in order to obtain gene expression profiles of embryos exposed to 5 Gy of gamma-rays compared to sham controls. For this purpose, total RNA was extracted using ISOGEN (Nippon Gene Co., Toyama, Japan) at 4 h after the gamma-ray irradiation of blastula embryos (*n* = 30), and purified with mini Quick Spin RNA Columns (Merck KGaA) according to manufacture instruction. RNA quality (RIN > 9) was assessed with Macrogen 2100 Bioanalyzer (Macrogen, Seoul, Korea). The RNA was sequenced (NovaSeq 6000) at Macrogen (Seoul, Korea). The RNA-seq data has been registered as the accession number DRA010906 in DDBJ database. Statistical analysis for detection of differentially expressed genes (DEG) was performed on a comparison pair (irradiated samples vs. sham controls) as requested using fragments per kilobase of transcript per million mapped reads (FPKM). DEG list was further analyzed with DAVID tool (http://david.abcc.ncifcrf.gov/) for gene set enrichment analysis per biological process.

### 2.6. Measurement of The Maximum Width of The Optic Tectum (OT)

To evaluate the reduction in brain size quantitatively post-irradiation, we measured the maximum width of the OT, which is the largest subdivision of the medaka brain. Previous our fining by histological analyses indicated that the measured value of it could be used as a useful tool for evaluation of brain size [4]. The fixed embryos with 4% paraformaldehyde in 0.1 M phosphate buffer for 1 day were mounted on a glass slide for measurement under a microscope (BX50, Olympus). Images were captured with a digital camera (DFC7000T, Leica).

### 2.7. Statistical Analysis

The maximum width of OT of gamma-ray irradiated or microbeam irradiated embryos were compared with those of sham-controls using one-way ANOVA and Dunnett’s multiple comparison test or the two-tailed student’s t test. Differences in the numbers of anti-P-H3-labeled cells between four or five irradiated embryos and sham-controls were examined using the two-tailed student’s *t* test. A *p* * value < 0.05 was considered statistically significant and *p* ** < 0.01 was considered highly statistically significant.

## 3. Results

### 3.1. Susceptibility of Embryos to Irradiation Decreased as the Development Proceeds During Extremely Early Embryogenesis Period

**Figure 1:**
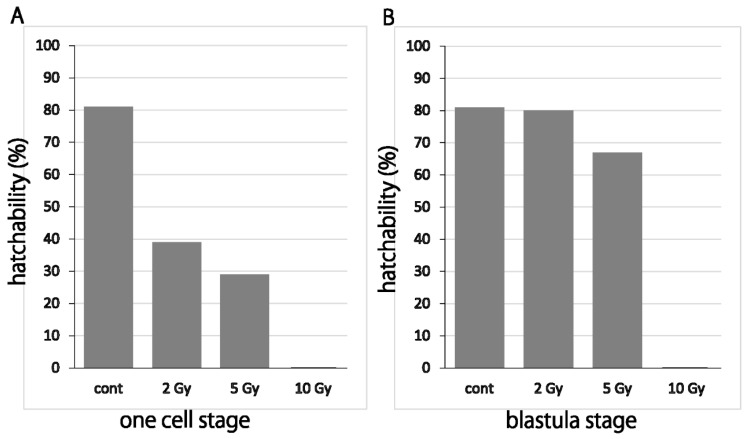
Susceptibility of embryos to irradiation decreased as the development proceeds in extremely early embryogenesis period. One cell stage embryos (**A**) and blastula stage embryos (**B**) were exposed to X-rays (2, 5 and 10 Gy) and gamma-rays (2, 5 and 10 Gy), respectively, and their hatchability were scored.

Only 39% of medaka embryos hatched normally when they were irradiated with 2 Gy of X-rays at one cell stage (30 min post-fertilization), while almost all (80%) embryos hatched normally when irradiated at blastula stage (6–8 h post-fertilization). The embryos which died before hatching showed abnormal development with malformations including abnormal vascular flow and severe body deformation (Table S1). When one cell stage embryos were irradiated with 5 Gy of X-rays, 29% of them hatched normally (Figure 1A and Table S1), whereas 67% of the embryos hatched normally, when irradiated with 5 Gy of gamma-rays at blastula stage (Figure 1B and Table S1). Since evaluations for exposure to X-rays and gamma-rays are considered as the same biological effects for their same radiation weighting factors [19], we don’t need to consider the difference of radiation quality between them. The susceptibility to irradiation of developing embryos was higher in the earlier stage and medaka embryos became more resistant to the damages induced by irradiation as the development proceeds.

### 3.2. Embryos Irradiated at Blastula Stage Showed Transient Delay of Brain Development but Normally Hatched

**Figure 2:**
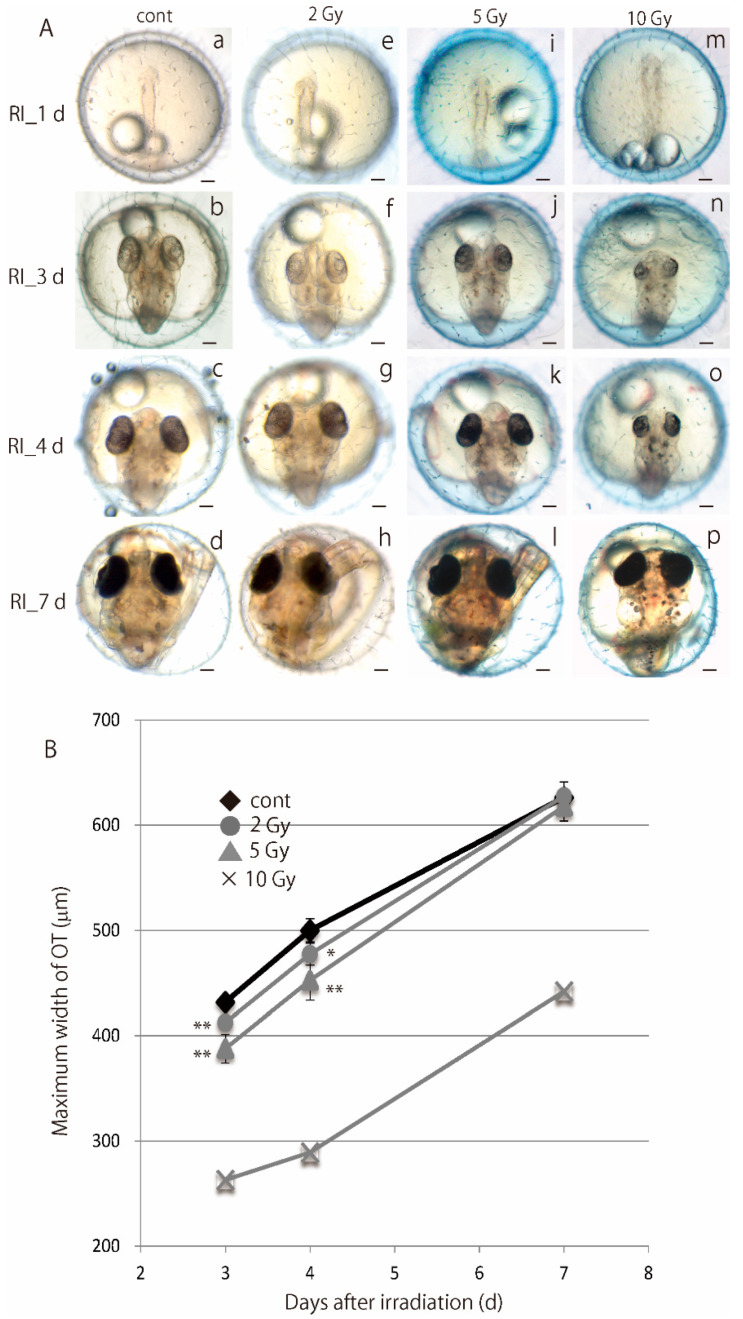
Transient delay of brain development before hatching in the irradiated blastula embryos with non-lethal dose of gamma-rays. Blastula embryos were irradiated with 2 or 5 Gy gamma-rays and hatched normally (h and l in **A**), whereas those irradiated with 10 Gy gamma-rays died before hatching due to severe malformations (*n*–*p* in **A**). At 3 days and 4 days after the irradiation, brains of the blastula embryos irradiated with 2 Gy or 5 Gy gamma-rays were smaller (f and j in **A**) than the controls (b in **A**). After that, the brains of the irradiated embryos developed rapidly and their size became equal to those of the control embryos at 7 days after the irradiation. The temporal changes of the maximum width of the OT were shown in (**B**). Error bars show S.D. **A** *p* * < 0.05 was considered statistically significant and *p* ** < 0.01 was considered highly statistically significant. Scale bars = 100 μm.

Medaka embryos irradiated with gamma-rays (2 and 5 Gy) at blastula stage developed to neurula stage without apparent abnormalities within 1 day after the irradiation (e in Figure 2A). Subsequently, almost all irradiated embryos with 2 Gy of gamma-rays (80% as shown in Figure 1A and Table S1) and a part of irradiated embryos with 5 Gy of gamma-rays which could hatch normally (67% as shown in Figure 1A and Table S1) exhibited developmental delay in the brain and their size (excluding lethal embryos at hatching) was significantly smaller (f and j in Figure 2A) than controls (b in Figure 2A): the maximum width of OT of the embryos irradiated with 2 and 5 Gy of gamma-rays were 412.5 ± 5.8 μm (mean ± SD) and 387.7 ± 13.7 μm (mean ± SD) at 3 days after the irradiation, 477.3 ± 10.4 μm (mean ± SD) and 452.5 ± 18.8 μm (mean ± SD) at 4 days after the irradiation, respectively. The maximum width of OT of controls at 3 and 4 days were 432.0 ± 5.2 μm (mean ± SD) and 500.0 ± 11.1 μm (mean ± SD), respectively. The maximum width of OT of the irradiated embryos with 2 and 5 Gy of gamma-rays at 3 days (*p* = 3.0 × 10^−3^, 4.2 × 10^−6^) and at 4 days (*p* = 2.0 × 10^−2^, 3.0 × 10^−4^) post-irradiation were significant smaller than their sham-controls by Dunnett’s multiple comparison test (Figure 2B).

After that, the brains of the embryos irradiated with gamma-rays (2 and 5 Gy) developed rapidly and their size (627.6 ± 13.8 μm, mean ± SD and 618.1 ± 14.2 μm, mean ± SD, respectively) became almost equal to those of the control embryos (626.6 ± 7.6 μm, mean ± SD) at 7 days after the irradiation with no statistical significance by one-way ANOVA (*p* = 0.5) (Figure 2B). The embryos irradiated with higher dose (10 Gy) of gamma-rays at blastula stage also developed to normal neurula with a slight retardation in development without induction of deformation compared to non-irradiated embryos (m in Figure 2A).

Irradiation-induced apoptotic cells were rarely found at 8 h after the gamma-ray irradiation (10 Gy) in the irradiated blastula embryos by histological analyses with H.E. stained sections (Figure S2). However, they all died before hatching with multiple developmental malformations such as abnormal vascular flow, numerous cell deaths in brain, microphthalmia and severe body deformation (n–p in Figure 2A).

### 3.3. Normal Embryonic Development Depends on the Proportion of Non-Damaged Cells in Blastoderm of Irradiated Embryo

**Figure 3:**
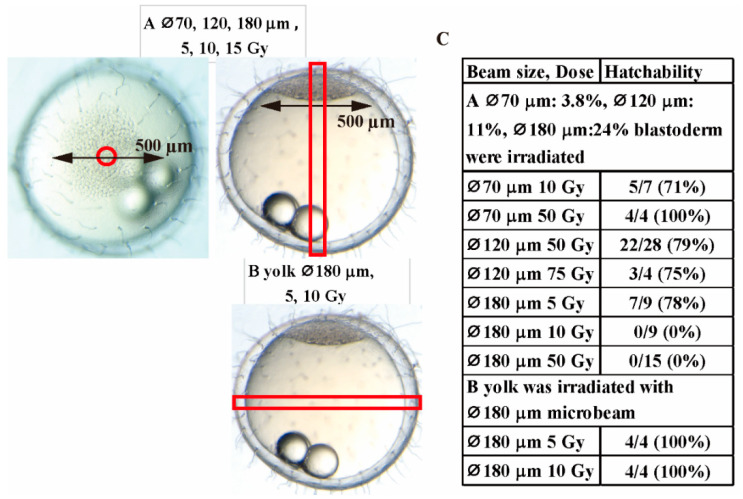
Targeted irradiation with a collimated carbon-ions microbeam on blastula embryo of medaka. The center part of blastoderm (diameter: 500 μm) was irradiated with a collimated carbon-ions microbeam with various diameters (70, 120, and 180 μm) and doses (5, 10, 50 and 75 Gy) (**A**). Yolk was irradiated with 5 and 10 Gy microbeam with 180 μm diameter (**B**). The targeting irradiated areas are surrounded by red line in the pictures. The hatchability of blastula embryos irradiated with microbeam are summarized in (**C**).

When the central part of blastoderm was irradiated with 70 and 120 μm diameter carbon-ion microbeam of 50 Gy, 3.8 and 11% of the blastoderm cells were irradiated, keeping all other cells of the blastoderm were not irradiated (Figure 3A), and 100 and 79% of the irradiated embryos developed and normally hatched, respectively (Figure 3C and Table S2). Irradiation with 120 μm diameter carbon-ion microbeam of 50 and 75 Gy induced similar disturbances of embryonic development: 79% and 75% of the embryos hatched normally (Figure 3C and Table S2), indicating that irradiation of carbon-ion microbeam higher than 50 Gy was enough to kill the irradiated cells in the blastoderm. These results suggest that the loss of approximately 10% or less of blastoderm cells can be compensated by the remaining cells during embryonic development.

**Figure 4:**
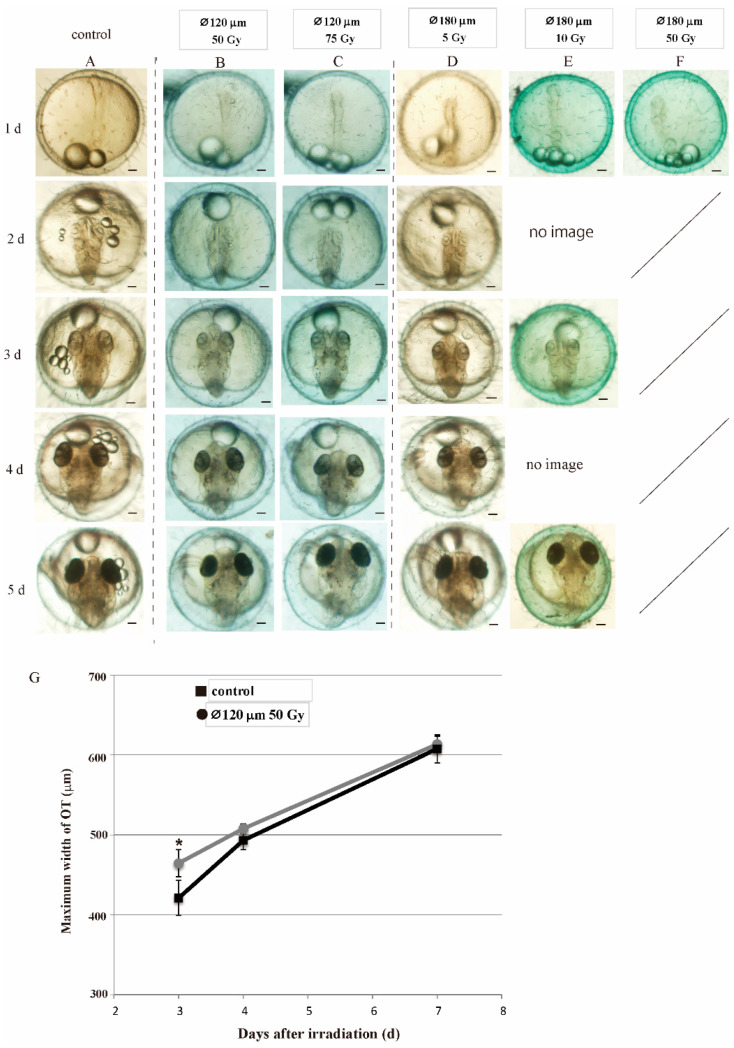
Targeted irradiation of the center of blastoderm with microbeam induced the same developmental disturbance as gamma-rays induced. Blastula embryos were irradiated with a collimated carbon-ions microbeam with diameter of 120 μm with 50 Gy (**B**) or 75 Gy (**C**), or a microbeam with diameter of 180 μm with 5 Gy (**D**) and all of the irradiated embryos hatched normally, whereas the embryos irradiated with a microbeam with diameter of 180 μm with 10 Gy died before hatching (**E**). At 3 days after the irradiation, brains of the blastula embryos irradiated with a microbeam with diameter of 120 μm with 50 Gy were significantly smaller than the controls (**A**,**B**,**G**). Subsequently, the brains of the irradiated embryos developed rapidly and their size became equal to those of the control embryos at 4 days and 7 days after the irradiation. The temporal changes of the maximum width of the OT were shown in **G**. Scale bars in (**A**–**F**) = 100 μm. Error bars show S.D. A *p* * value < 0.05 was considered statistically significant.

Fifty and 75 Gy carbon-ion microbeam with 120 μm diameter and 5 Gy microbeam with 180 μm diameter induced transient delay of brain development at 3 and 4 days after the irradiation (Figure 4B–D,G and Figure S3) compared to sham-controls (Figure 4A), as 2 and 5 Gy gamma-ray irradiation induced in the medaka embryos (Figure 2). The maximum width of OT of the microbeam (⌀120 μm, 50 Gy) irradiated embryos at 3 days after the irradiation was 421.1 ± 27.0 μm (mean ± SD, *n* = 5) and significantly smaller than the non-irradiated controls (464.7 ± 17.0 μm, mean ± SD, *n* = 5) by two-tailed student’s *t* test (*p* = 0.049). Subsequently, the brain of the irradiated embryos developed rapidly (493.3 ± 12.9 μm, mean ± SD, *n* = 5) and grew to almost same size as the controls (508.0 ± 5.8 μm, mean ± SD, *n* = 5) at 4 days after the irradiation. Thereafter, the maximum width of the OT of the microbeam irradiated embryos was 607.9 ± 19.2 μm (mean ± SD) and almost equal to that of control embryos (613.6 ± 10.0 μm, mean ± SD) at 7 days after the irradiation (Figure 4G). The loss of approximately 10% of the blastoderm cells resulted in the normal embryonic development except the transient developmental delay in the brain, as gamma-ray irradiation of low dose (2 and 5 Gy) induced in medaka embryos.

When the central part of blastoderm was irradiated with 180 μm diameter microbeam of 50 Gy, killing 24% of the blastoderm cells, all of the irradiated embryos also developed normally to neurula stage within 1 day after the irradiation (Figure 4F). After that, the embryos showed the same developmental defects such as abnormal vascular flow, numerous cell deaths in the brain, microphthalmia and severe body deformation, thereafter they all died before hatching, suggesting that the loss of about 25% cells of blastoderm could not be compensated by the remaining cells in the blastoderm and the irradiated embryos failed to develop further than neurula stage. When blastoderm was irradiated with 180 μm diameter microbeam of 10 Gy, the irradiated embryos showed the same developmental malformations as those induced by irradiation with 10 Gy gamma-rays, such as abnormal vascular flow, numerous cell deaths in brain, microphthalmia, severe body deformation (Figure 4E) and none of the irradiated embryo could hatch (Figure 3C).

When only yolk was irradiated with 180 μm diameter microbeam with 5 Gy (*n* = 4) and 10 Gy (*n* = 4) as shown in Figure 3B, all of the irradiated embryos developed and hatched normally with no apparent abnormalities (hatching rate: 4/4 and 4/4). These results of targeted irradiation using collimated microbeam demonstrate that gamma-rays and carbon-ions induced the same disturbance in medaka embryonic development, depending on dose and size of irradiated area, respectively.

### 3.4. Irradiated Blastoderm Cells Did Not Arrest Cell Cycle Progression

**Figure 5:**
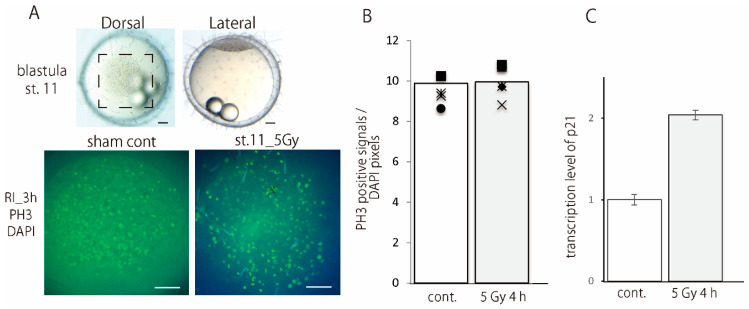
Irradiated blastoderm cells did not arrest cell cycle progression. Anti-phospho-histone H3 (P-H3)-positive cells were counted at 4 h after the irradiation of gamma-rays. The P-H3 positive signals in the blastoderm (squared with dotted lines in (**A**)) of irradiated embryos at stage 11 (st11_5Gy) and sham control (sham cont) are shown in dorsal view in the lower row. Each data was shown with different markers in (**B**). There was not significant difference in the number of anti-P-H3 positive cells between the irradiated (*n* = 5) and control embryos (*n* = 4) (**B**). Quantitative real-time PCR demonstrated that p21 expression was up-regulated with twice higher in the irradiated blastula embryos at 4 h after the irradiation (**C**). Scale bars in A = 100 μm. Error bars show S.D.

There was no significant difference between the number of anti-P-H3-positive cells at 4 hr after the gamma-ray irradiation (5 Gy) in the irradiated blastula embryos (9.9 ± 0.8, mean ± SD, *n* = 5) and the sham controls (9.4 ± 0.7, mean ± SD, *n* = 4) (*p* = 0.29 by two-tailed student’s t test) (Figure 5A,B). Transcription of cyclin-dependent kinase inhibitor 1A, p21, was up-regulated to twice in comparison to the non-irradiated control at 4 h after the irradiation (Figure 5C). The transcriptome analysis confirms that p21 was up-regulated significantly (3.62 fold change) in the irradiated blastulas at 4 h after the irradiation (Dataset S1), however, it was also demonstrated that the genes responding to DNA damage, those annotated into intrinsic apoptosis, nuclear division, DNA repair and cell-cycle, did not change significantly due to within the fold change ≤±1.5 in the irradiated blastula embryos at 4 h after irradiation (Table 1, Dataset S1).

### 3.5. Gamma-Ray Irradiation on the Embryos at Late Embryogenesis Period Resulted in Incomplete Development of Brain

**Figure 6:**
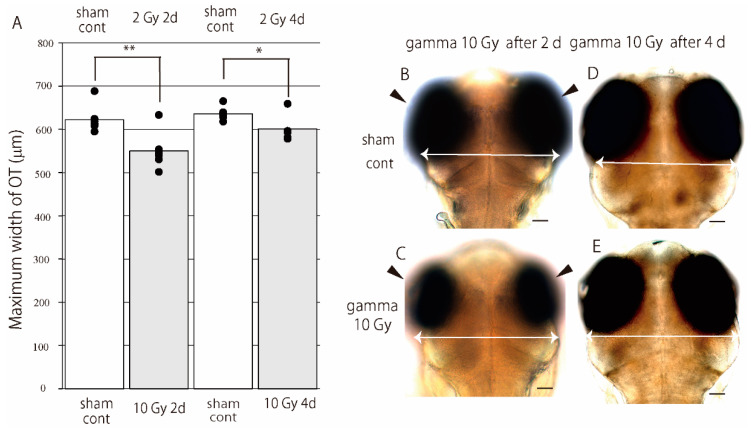
Gamma-ray irradiation on the embryos at late embryogenesis period resulted in the incomplete development of brain. Medaka embryos at 3 dpf were irradiated with 10 Gy gamma-rays and maximum width of OT were measured at 2 and 4 days after the irradiation (**A**). Each independent data was shown with black circled marker in A. At 2 days after the irradiation, the maximum width of OT of the irradiated embryos (**C**) were smaller than the non-irradiated sham controls (**B**) with a statistical significance. At 4 days after the irradiation, OT of the irradiated embryos (**E**) were still smaller than the non-irradiated sham controls (**D**) with a statistical significance. Retina of the irradiated embryos were also smaller than the sham controls at 2 days after the irradiation (arrowheads in **B**,**C**). All of the irradiated embryos hatched normally. A *p* * value < 0.05 was considered statistically significant and *p* ** < 0.01 was considered highly statistically significant. Scale bar = 50 μm.

Embryos at the late embryogenesis period, at 3 days post-fertilization (3 dpf), were irradiated with 10 Gy of gamma-rays. The maximum width of their OT were measured at 2 and 4 days after the irradiation. At 2 days after the irradiation, the maximum width of OT of the irradiated embryos (Figure 6C) were smaller than the non-irradiated sham controls (Figure 6B) with a statistical significance by two-tailed student’s t test (*p* = 0.003) (Figure 6A). At 4 days after the irradiation (at hatching), OT of the irradiated embryos (Figure 6E) were still smaller than the non-irradiated sham controls (Figure 6D) with a statistical significance by two-tailed student’s t test (*p* = 0.018). Retina of the irradiated embryos were also smaller than the sham controls at 2 days after the irradiation (arrowheads in Figure 6B,C). All of the irradiated embryos with 10 Gy gamma-rays could hatch normally.

## 4. Discussion

### 4.1. The Responses of Medaka Blastula Embryos after Irradiation Were Similar to Those of Pre-Implantation Period Embryos of Mammals

In this study, almost all irradiated embryos with low dose of gamma-rays with 2 Gy and a part of irradiated embryos with 5 Gy of gamma-rays which could hatch normally exhibited a transient delay of brain development. When the blastula embryos irradiated with higher dose of gamma-rays (10 Gy), the embryos developed normally up to neurula stage and died before hatching with severe developmental malformations. Collectively, we found here that irradiation of blastula embryos of medaka resulted in the low occurrence of teratogenicity in the hatched larvae. Since blastula stage embryos of mammals are pre-implantation, these responses of medaka embryos are interestingly similar to those of mammalian embryos where the teratogenicity in the offspring is low when the embryos are exposed to IR during the pre-implantation period [1,2,3]. These findings suggest a possibility that the low teratogenicity of mammalian embryos irradiated during the pre-implantation period might not be caused by promoted abortion of the injured embryos, but by repairing IR induced damages with the high pluripotency of endoderm cells in the irradiated embryos. The further study of irradiation on the embryonic development in oviparous fish such as medaka and zebrafish can be highly helpful to understand the biological processes of IR responses of mammalian embryos in utero and why the damaging effects of irradiation on embryonic development depends on gestational age when radiation exposure occurs.

### 4.2. The Developmental Disturbance in IR Irradiated Embryos Might Be Determined by the Proportion of Severely Injured Cells

To investigate the machinery for restoration of irradiation-induced disturbance of embryonic development in medaka, we conducted targeted irradiation of blastoderm using a collimated carbon-ion microbeam with diameters of 70, 120 and 180 μm in TIARA, QST [9,10]. One of the most important findings in this study is that the microbeam irradiation of small area of blastoderm mimics the low dose gamma-ray irradiation and the irradiation of large area of blastoderm mimics the high dose gamma-ray irradiation. This finding strongly suggests that the developmental disturbance in the IR-irradiated embryos is determined by the proportion of severely injured cells or remaining intact cells in the irradiated blastoderm. Targeted irradiation provides the first direct evidence to explain how IR irradiation disturbs embryonic development through damages in individual cells.

The severely injured cells in the gamma-ray irradiated blastoderm would not contribute embryonic development since they would die or be senescent to stop proliferation. If the cells with minor damage took parts in the following development, it might be increasing the risk of teratogenesis. Since it is unlikely that all of the injured cells died or were repaired perfectly, there should be a machinery to avoid the participation of the injured blastoderm cells during embryogenesis. In mouse embryo, cell competition occurs between cells with different Myc expression levels in epiblast and it is proposed that cell competition-mediated phenomenon may act as a homeostatic monitoring system that eliminates defective cells in early period of embryogenesis [20,21,22]. Cell competition in embryonic development has been first discovered in Drosophila [23] and it is very likely that the same mechanism is shared in fish embryos. The minor injured cells by irradiation in the medaka blastula embryos might be removed not to participate during embryogenesis through the cell competition-mediated phenomenon.

### 4.3. Brain Development Delayed Transiently in Irradiated Blastula Embryos Whereas the Irradiated Embryos during Late Embryogenesis Peiod Resulted in Incoplete Brain Development

When blastula embryos were exposed to gamma-rays with low dose (2 and 5 Gy) or about 10% or less of them were irradiated with 50 Gy of carbon-ion microbeam, they hatched normally even though they showed a transient delay of brain development before hatching. In medaka, the pluripotent ability of blastoderm cells has been identified in vitro and in vivo [6,7,8,24] and the loss of about 10% or less of blastoderm cells might be compensated by the remaining non-damaged pluripotent blastoderm cells. In contrast, the loss of about 25% of the blastoderm cells resulted in the lethality before the time of hatching, suggesting that there is an upper limit of proportion of the lost cells to compensate and restore the development of irradiated embryos.

Exposure to IR at post-organogenesis period (embryonic day 13.5) increased the risk of brain abnormalities such as microcephaly in mouse [25,26]. Among the atomic bomb survivors in Hiroshima and Nagasaki, microcephaly was reported in those who exposed to IR in utero during 8–15 weeks post-ovulation [3,27].

The most well-developed brain part in mammals is the cerebrum, whereas that in the medaka is the optic tectum in the midbrain. These two species have different structures of the brain in adults; however, they share the common conserved regulation for morphogenesis and regionalization in their embryonic brains [28]. When the medaka embryos at 3 dpf were exposed to X-rays, the brain was markedly smaller than the control at the time of hatching [4]. Additionally, we report here a similar finding that the brains were significantly smaller than the sham-controls at the time of hatching when the medaka embryos at 3 dpf were exposed to gamma-rays (Figure 6). These findings suggest that brain development is the most susceptible process to IR-irradiation during embryogenesis in vertebrates and that the loss of pluripotent capacity in the embryonic cells as the development proceeds is associated with the increase of malformation in the embryos irradiated during later period of embryogenesis.

### 4.4. Blastula Embryo Which Was Irradiated with a Lethal Dose IR Can Develop up to Neurula

It is a noteworthy finding of this study that the embryos developed normally up to neurula, thereafter they all died due to severe malformations before hatching, when blastula embryos were irradiated with a lethal dose of IR (gamma-rays of 10 Gy or about 25% of blastoderm cells were irradiated with 50 Gy of microbeam). When morula stage (stage 8) medaka embryos were irradiated, no apoptotic cells were found at 30 min after the irradiation, whereas 24% of irradiated embryos exhibited apoptotic cells when irradiated at pre early gastrula stage (stage 12) [29]. We preliminarily observed the cell movement of the irradiated blastula stage embryos (stage 11) by time-lapse video microscopy for several hours after the targeted irradiation and did not observe any abnormal cell movement in the irradiated blastoderm [30]. Previous studies in medaka demonstrated that the time point of the start of zygotic gene transcription, mid-blastula transition (MBT), was around stage 11 [31,32]. In zebrafish and *Xenopus*, the embryos before MBT lack the ability to arrest cell cycle progression by checkpoint and they acquire the ability to perform apoptosis after MBT [24,33,34,35,36,37]. Our transcriptome analysis in medaka supports these findings, since the genes related with apoptotic induction, cell proliferation, DNA repair and cell cycle arrest might not fully function in the irradiated blastula at 4 h after the irradiation (Table 1 and Dataset S1), which provides the evidence that blastoderm cells do not arrest cell cycle even when they are severely damaged by exposure to high-dose IR. It might be noteworthy that the expression of p21, which suppresses cyclin/CDK activity and induced cell cycle arrest [38,39], was up-regulated in the irradiated blastula embryos of medaka. Since senescent cells are expressing high levels of p21 [40,41], it would be possible that severely damaged cells in the irradiated embryos were in cellular senescence, which do not show up-regulation of genes relating with DNA repair and cell cycle arrest, instead of apoptotic incidence [42,43]. Similar findings were also reported in mice and human embryonic stem cells, that p21 mRNA was up-regulated following DNA damage but P21 protein was not translated [44,45,46], which requires more detailed analyses to understand our incidence.

## 5. Conclusions

In this study, we investigated the effects of IR exposure at blastula stage in medaka embryos and demonstrated that the responses of medaka blastula embryos after irradiation were similar to those of pre-implantation period embryos of mammals. We also conducted targeted irradiation of blastoderm using a collimated carbon-ion microbeam in TIARA, QST, and found for the first time that the proportion of severely damaged cells or remaining intact cells in the irradiated blastoderm determines the success or failure of embryonic development. Brain development in vertebrate embryos might be the most susceptible process to IR in embryogenesis and the loss of pluripotent capacity in the embryonic cells as the development proceeds could be associated with the increase of malformation in the irradiated embryos at late embryogenesis period. It is also noteworthy that the embryos developed normally up to neurula even when the embryos were irradiated with IR at a dose far exceeding the lethal level. Medaka is a useful model of mammals to study the effects of IR in early embryogenesis period and the findings in this study may open an avenue to understand why IR-induced teratogenesis would be rare incidence in contrast with the high frequency of lethality when exposed to IR at pre-implantation period in mammals.

## Figures and Tables

**Table 1:** Intrinsic apoptotic signaling pathway in response to DNA damage by p53 mediator.

Gene ID	Gene Name	Discription	Fold Change IR Embryos/Sham-Cont.
ENSORLG00000016085	hipk2	homeodomain interacting protein kinase 2 [Source:ZFIN;Acc:ZDB-GENE-031125-4]	−1.40
ENSORLG00000010268	msh2	mutS homolog 2 (E. coli) [Source:ZFIN; Acc:ZDB-GENE-040426-2932]	−1.32
ENSORLG00000016313	si:ch211-160o17.4	si:ch211-160o17.4 [Source:ZFIN; Acc:ZDB-GENE-141215-36]	−1.15
ENSORLG00000022951	si:dkey-74k8.3	si:dkey-74k8.3 [Source:ZFIN; Acc:ZDB-GENE-141222-20]	−1.07
ENSORLG00000012316	not defined	homeodomain-interacting protein kinase 3 [Source:NCBI gene;Acc:101162288]	1.06

## Supplementary Materials

The following are available online at https://www.mdpi.com/2079-7737/9/12/447/s1, Figure S1: The carbon-ion tracks of the collimated microbeams used for the targeted irradiation of blastula stage embryos, Figure S2: Histological analysis in irradiated blastula embryos with high dose of gamma-rays at 8 h after irradiation, Figure S3: Sequences of embryonic development for 7 days following the targeted irradiation using a collimated carbon-ions microbeam of 50 Gy with diameter of 120 μm, Table S1: Hatchability of the one cell stage embryos irradiated with X-rays and the blastula stage embryos irradiated with gamma-rays, Table S2: Hatchability of blastula stage embryos irradiated with different size and dose of carbon-ion microbeam, Dataset S1: the genes responding to DNA damage, those annotated into intrinsic apoptosis, nuclear division, DNA repair and cell-cycle.
