# Supplementary material for: Collimated Microbeam Reveals that the Proportion of Non-Damaged Cells in Irradiated Blastoderm Determines the Success of Development in Medaka (Oryzias latipes) Embryos
Source: Biology (Basel). 2020 Dec 5;9(12):447. doi: 10.3390/biology9120447 (PMC7762064; doi:10.3390/biology9120447)
Supplement: Supplementary file 1 [file biology-09-00447-s001.zip › biology-970353-suppl final/Supplemental data/biology-970553-suppl-proof_TY.docx]

Article

Collimated Microbeam Reveals that the Proportion of Non-Damaged Cells in Irradiated Blastoderm Determines the Success of Development in Medaka (*Oryzias latipes*) Embryos

Takako Yasuda, Tomoo Funayama, Kento Nagata, Duolin Li, Takuya Endo, Qihui JIA, Michiyo Suzuki, Yuji Ishikawa, Hiroshi Mitani and Shoji Oda


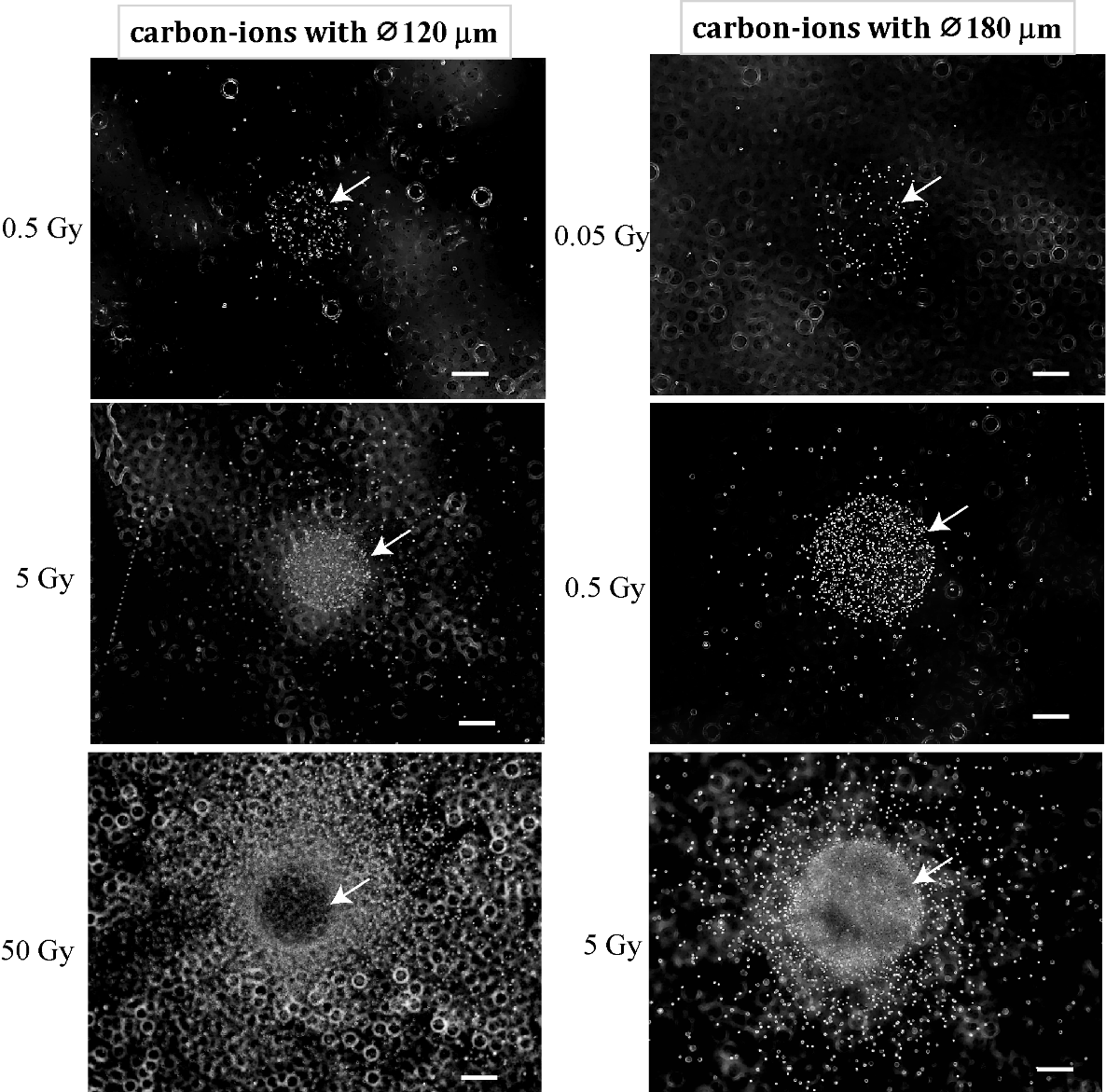


**Figure 1.** The carbon-ion tracks of the collimated microbeams used for the targeted irradiation of blastula stage embryos. The carbon-ion tracks of the collimated microbeams with diameter of 120 μm and doses of 0.5, 5 and 50 Gy and with diameter of 180 μm and doses of 0.05, 0.5 and 5 Gy were visualized on CR39 membrane by etching. Scale bars = 50 μm.


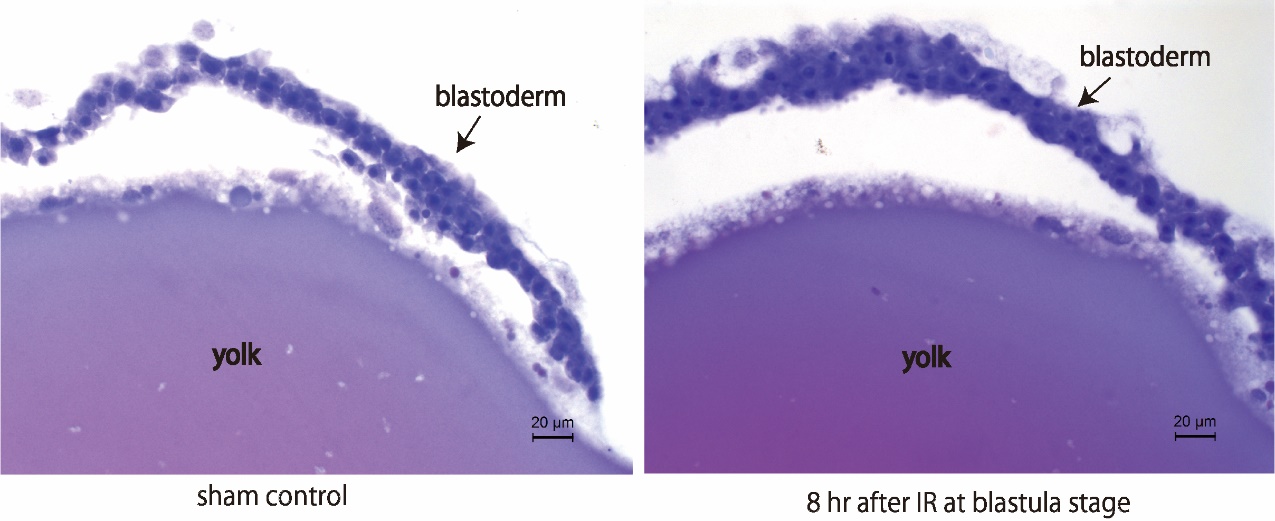


**Figure 2.** Histological analysis in irradiated blastula embryos with high dose of gamma-rays at 8 hr after irradiation. Histological analyses by H.E. stained section showed that irradiation-induced apoptotic cells were rarely found at 8 hr after the gamma-ray irradiation (10 Gy) in the irradiated blastoderm cells (B) compared to sham-control (A). Scale bars = 20 μm.


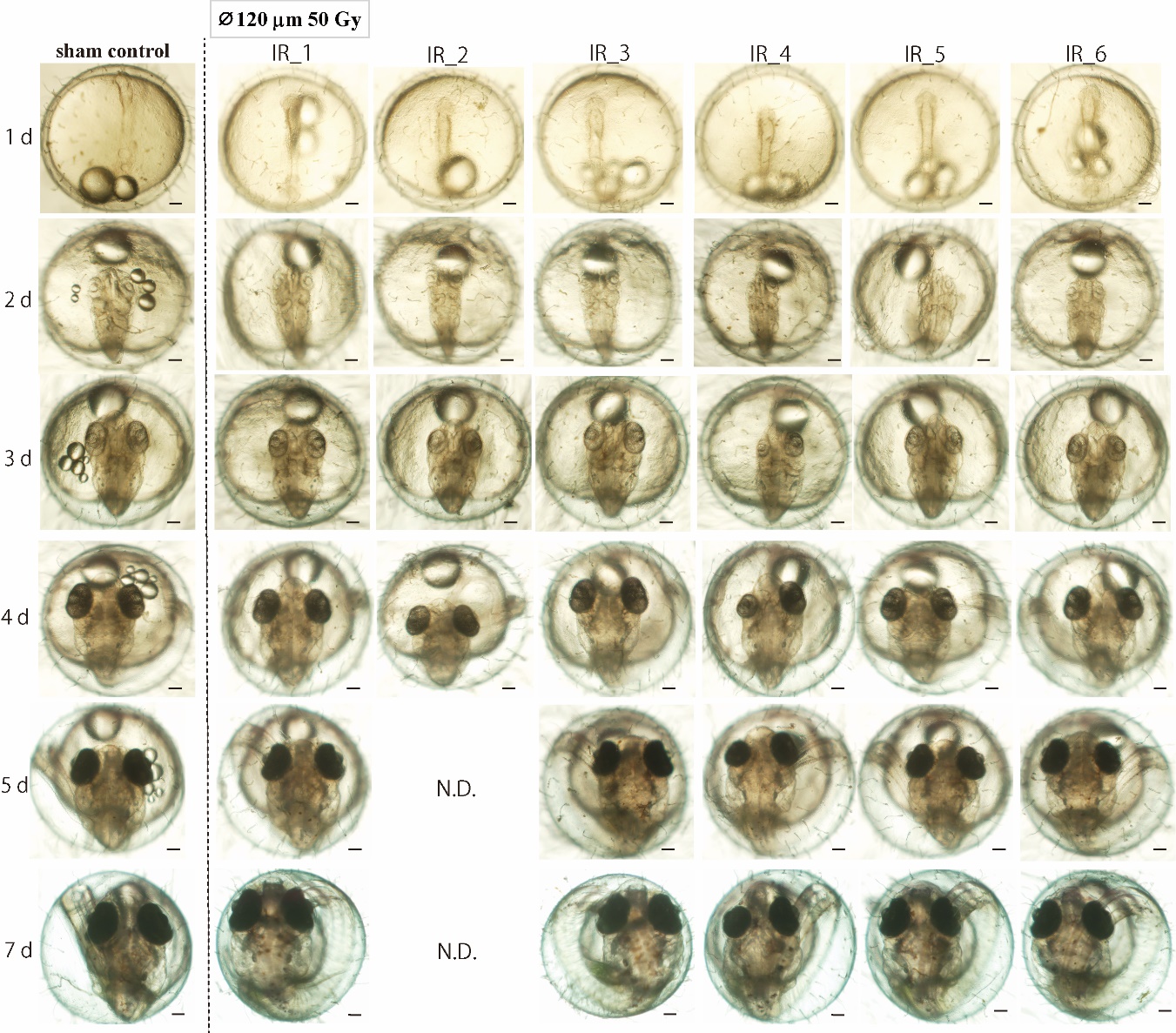


**Figure 3.** Sequences of embryonic development for 7 days following the targeted irradiation using a collimated carbon-ions microbeam of 50 Gy with diameter of 120 μm. Within the embryos irradiated with a collimated carbon-ions microbeam with 120 μm diameter and dose of 50 Gy (*n* = 28), typical 6 embryos are shown. The brains of the irradiated embryos were smaller than the non-irradiated controls only during 3 and 4 days after the irradiation. Thereafter, the brains rapidly developed to the same size as the non-irradiated controls. Scale bars = 50 μm.

**Table S1.** Hatchability of the one cell stage embryos irradiated with X-rays and the blastula stage embryos irradiated with gamma-rays.

| **One Cell Stage** | | | | | | |
| --- | --- | --- | --- | --- | --- | --- |
| **Dose** | **No. Embryos** | **Embryonic Death (St.16)** | **Embryonic Death (Before Hatching)** | **Abnormal Hatching** | **Normal Hatching** | **Hatchability (%)** |
| 0 | 172 | 11 | 13 | 9 | 139 | 81 |
| 2 | 118 | 11 | 47 | 14 | 46 | 39 |
| 5 | 126 | 19 | 53 | 18 | 36 | 29 |
| 10 | 113 | 38 | 75 | 0 | 0 | 0 |
| Blastula Stage | | | | | | |
| **Dose** | **No. Embryos** | **Embryonic Death (St.16)** | **Embryonic Death (Before Hatching)** | **Abnormal Hatching** | **Normal Hatching** | **Hatchability (%)** |
| 0 | 48 | 4 | 3 | 2 | 39 | 81 |
| 2 | 50 | 6 | 1 | 3 | 40 | 80 |
| 5 | 65 | 7 | 5 | 9 | 44 | 67 |
| 10 | 48 | 3 | 45 | 0 | 0 | 0 |

**Table S2.** Hatchability of blastula stage embryos irradiated with different size and dose of carbon-ion microbeam.

| **Microbeam Size and Irradiation Dose (Gy)** | **No. Embryos** | **Embryonic Death (St.16)** | **Embryonic Death (Before Hatching)** | **Abnormal Hatching** | **Normal Hatching** | **Hatchability (%)** |
| --- | --- | --- | --- | --- | --- | --- |
| ⌀70 mm 10 Gy | 7 | 0 | 2 | 0 | 5 | 71 |
| ⌀70 mm 50 Gy | 4 | 0 | 0 | 0 | 4 | 100 |
| ⌀120 mm 50 Gy | 28 | 0 | 4 | 2 | 22 | 79 |
| ⌀120 mm 75 Gy | 4 | 0 | 1 | 0 | 3 | 75 |
| ⌀180 mm 5 Gy | 9 | 0 | 1 | 1 | 7 | 78 |
| ⌀180 mm 10 Gy | 9 | 0 | 8 | 1 | 0 | 0 |
| ⌀180 mm 50 Gy | 15 | 0 | 6 | 0 | 0 | 0 |
| yolk ⌀180 mm 5 Gy | 4 | 0 | 0 | 0 | 4 | 100 |
| yolk ⌀180 mm 10 Gy | 4 | 0 | 0 | 0 | 4 | 100 |

Note: Blastula embryos were exposed to carbon-ion microbeam.

**Publisher’s Note:** MDPI stays neutral with regard to jurisdictional claims in published maps and institutional affiliations.

| 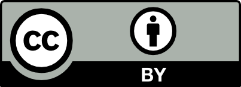 | © 2020 by the authors. Licensee MDPI, Basel, Switzerland. This article is an open access article distributed under the terms and conditions of the Creative Commons Attribution (CC BY) license (http://creativecommons.org/licenses/by/4.0/). |
| --- | --- |
